# Prevalence and anatomical variations of maxillary sinus septa: A cone-beam computed tomography analysis

**DOI:** 10.4317/jced.59599

**Published:** 2022-09-01

**Authors:** Farida Abesi, Samaneh Motaharinia, Ehsan Moudi, Sina Haghanifar, Soraya Khafri

**Affiliations:** 1 Dental Materials Research Center, Department of Oral and Maxillofacial Radiology, Dental Faculty, Babol University of Medical Sciences, Babol, Iran; 2 Student Research Committee, Babol University of Medical Sciences, Babol, Iran; 3 Department of Oral and Maxillofacial Radiology, Dental Faculty, Babol, Iran; 4 Social Medical and Health Department, Babol University of Medical Sciences, Babol, Iran

## Abstract

**Background:**

Detailed understanding the anatomy and variations of maxillary sinus septa is necessary. We aimed to investigate prevalence and various anatomical variations of maxillary sinus septa using cone-beam computed tomography (CBCT) images.

**Material and Methods:**

In this retrospective cross-sectional study, CBCT scans of 809 patients referred to a specialist dental and maxillofacial radiology clinic in Babol, northern Iran, during 2014-2020 were evaluated. Location, morphology, height, and direction of septa in different planes were recorded.

**Results:**

Sinus septa were observed in 37.9% (n=307) of the participants. There were 117 cases with septa only in left sinus, 83 cases with septa only in right sinus, and 107 cases with septa in both sinuses. Totally, 414 septa were seen in the subjects. Prevalence of partial septa was considerably higher than complete septa (77.5% versus 22.5%). Regarding septa location, middle area with a prevalence rate of 58.7% was found more prevalent than other sinus areas. The mean septum height on the left and right sinuses was 6.0±3.1 mm 5.7±3.9, respectively (*p*<0.001). Regarding left sinus, directions of vertical (63.8%), oblique (30.8%), and straight (63.8%) were the most prevalent compared with other directions in sagittal, frontal, and axial planes, respectively. In relation to right maxillary sinus, direction of vertical in sagittal and frontal planes (72.6% and 24.2%, respectively), and direction of straight in axial plane (58.4%) were the most prevalent directions compared with others in the same planes.

**Conclusions:**

Septa prevalence in maxillary sinus was relatively high, with various directions, locations, heights, and shapes. It is recommended to use CBCT for a careful evaluation of toothless area prior to surgeries on the sinus (especially sinus lift) in three plans, in order to take careful measures and prevent postoperative complications in case septa exist.

** Key words:**Maxillary sinus septa, anatomical variation, cone-beam computed tomography, dental implant.

## Introduction

The presence of septa should be considered for any maxillary sinus surgeries, because the septa can lead to perforation of the Schneiderian membrane (the unique lining of the nasal cavity and paranasal sinuses) (1,2). They can also complicate the procedures of sinus lift or implant surgery when their height is high. Furthermore, in case of complete septa, they can cause increase in sinus discharge or opacification, leading to a measurement error due to mistakenly seeing double sinus floor. Moreover, septa are sometimes misdiagnosed with cysts (3,4).

Panoramic imaging is a commonly used radiographic technique in dentistry, but septa cannot be observed in all images, because it is a two-dimensional radiography and some kinds of septa are not found in frontal plane and it is necessary to check sagittal and axial planes for detecting them (5).

Cone-beam computed tomography (CBCT) is a high-resolution three-dimensional imaging method that can prepare any desired imaging planes. It is also possible to exactly measure the dimensions and angles of septa by CBCT (6,7). Moreover, the detection of sinus pathology by CBCT has been stated to be more accurate than panoramic radiography (6,7).

Considering that the maxillary sinus lift procedure and use of implant has become popular, detailed understanding the maxillary sinus anatomy and its variations seems necessary (8,9). This knowledge can also help in differentiation of normal variations and pathologic lesions such as cysts, tumors, polyps, mucous retention cyst, etc. (10). The aim of this study was to investigate the prevalence and various anatomical variations of the maxillary sinus septa using CBCT images.

## Material and Methods

In this retrospective cross-sectional study, CBCT scans of patients referred to a specialist dental and maxillofacial radiology clinic in Babol, northern Iran, during 2014-2020 were evaluated. The CBCT images had to be acquired using the same scan settings and in the same CBCT unit. The exclusion criteria were as follows: 1) Individuals aged <18 years old; 2) Partial or incomplete images; 3) A history of maxillary surgery or acute trauma; 4) Lesions in maxillary sinus; 5) Septa of ≤2.0 mm.

All CBCT scans were obtained using Newtom Giano (Verona, Italy) machine with the following parameters: 90 KvP; 10 mAs; focal spot = 0.05 mm; scan time = 18-26 s; x-ray emission time = 3.6 s; axial thickness = 0.300 mm; field of view = 11 × 13 cm. CBCT images were then investigated and the following information were recorded in a checklist: septum location, septum morphology, septum height, septum direction in different planes. The presence or absence of the maxillary sinus septa was determined using the sagittal, frontal, and axial planes. The septum location was divided into three areas, including anterior (from mesial to second premolar), middle (from distal to second premolar to second molar), and posterior (from distal to second molar) (11). To evaluate the septum direction, the angle with the vertical axis was measured in each plane (sagittal, frontal, and axial); then, the septum direction was defined as vertical (<30°), oblique (between 30° and 60°), and straight (between 60° and 90°). In terms of morphology, septum was considered as ‘complete’ if it originated from a sinus wall and extended to reach the opposing sinus wall, in two or three planes; if the septum did not reach the opposing wall, it was considered as ‘partial’. To assess the septum height, a line was drawn at the approximate base of the septum to the highest point.

All statistical analyses were performed using SPSS v22. Descriptive analysis was used to calculate the mean and standard deviation for the continuous variables. Student t-test was used to compare the septum height between the left and right sinuses. The chi-squared test was also used to compare the categorical variables (such as sex, age groups, tooth loss, and septa location, morphology, height, and direction). A *p-value* less than 0.05 was considered significant.

We obtained a written consent form from all participants. The study protocol was approved by the ethics committee of Babol University of Medical Sciences (code: MUBABOL.REC.1395.222). The patients’ information was kept confidential.

## Results

A total of 809 subjects were enrolled in the study, of whom 378 (46.7%) were men and 431 (53.3%) were women. The mean age was 45.7±12.2, ranging from 18 to 76 years old. Sinus septa were observed in 37.9% (n=307) of the participants. There were 117 cases with septa only in the left sinus, 83 cases with septa only in the right sinus, and 107 cases with septa in both sinuses. Out of 307 subjects with maxillary sinus septa, 160 (42.3%) were men and 147 (34.1%) were women, and the difference was significant (*p*=0.016). The septa were also found in cases younger than 40 years (41.7%) more than in those aged 40-59 years (38.1%) and ≥60 years (26.8%).

**Figure 1:**
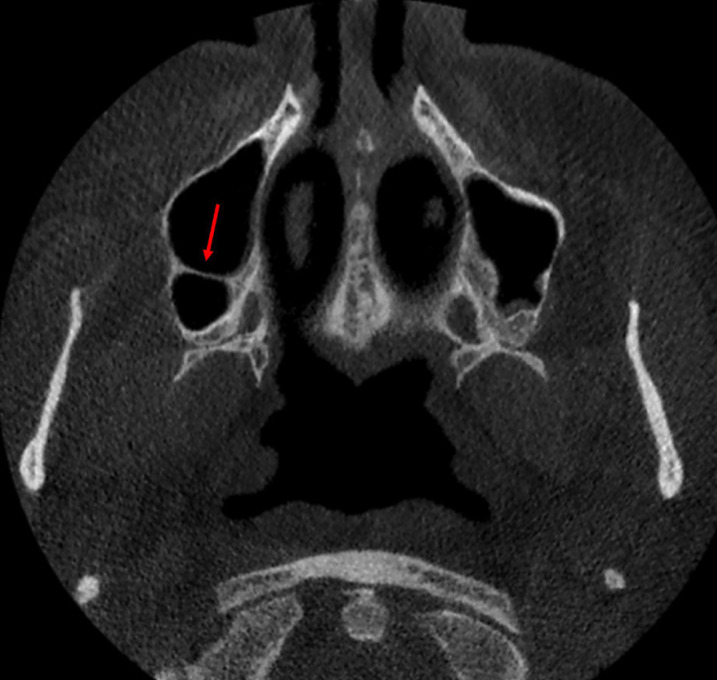
Axial view of cone-beam computed tomography showing a complete septum in the posterior area of the right maxillary sinus.

**Figure 2:**
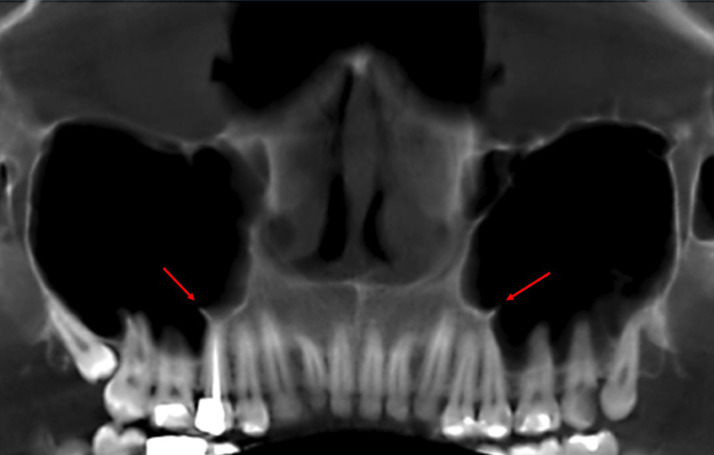
Panoramic-like view of cone-beam computed tomography showing bilateral partial septa.

In total, 414 septa were seen in the subjects. Assessment of septa morphology indicated that prevalence of partial septa was considerably higher than complete septa (77.5% versus 22.5%). Regarding septa location, middle area with a prevalence rate of 58.7% was found more prevalent than anterior (31.2%) and posterior (27.5%) locations. The mean septum height for all septa was 5.9±3.6 mm, ranging from 2.1 to 24.5 mm; the mean septum height on the left and right sinuses was 6.0±3.1 mm 5.7±3.9, respectively, with a significant difference (*p*<0.001). In Table 1, details of the abovementioned data have separately been reported for the left and right sinuses. Also, septa of some cases have been shown in Figs. 1,2.

**Table 1:**
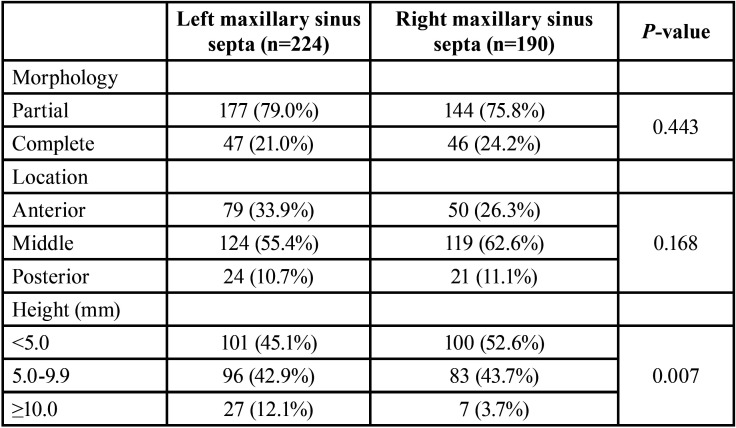
Morphology, location, and height of maxillary sinus septa.

The distribution of septa direction in various planes was separately represented for the left and right maxillary sinuses in Table 2. Regarding the left sinus, the directions of vertical (63.8%), oblique (30.8%), and straight (63.8%) were the most prevalent compared with other directions in the sagittal, frontal, and axial planes, respectively. In relation to the right maxillary sinus, the direction of vertical in the sagittal and frontal planes (72.6% and 24.2%, respectively), and the direction of straight in the axial plane (58.4%) were the most prevalent directions compared with others in the same planes; of course, it is noteworthy that the direction of most of the septa (40.0%) was not observed in the frontal plane.

**Table 2:**
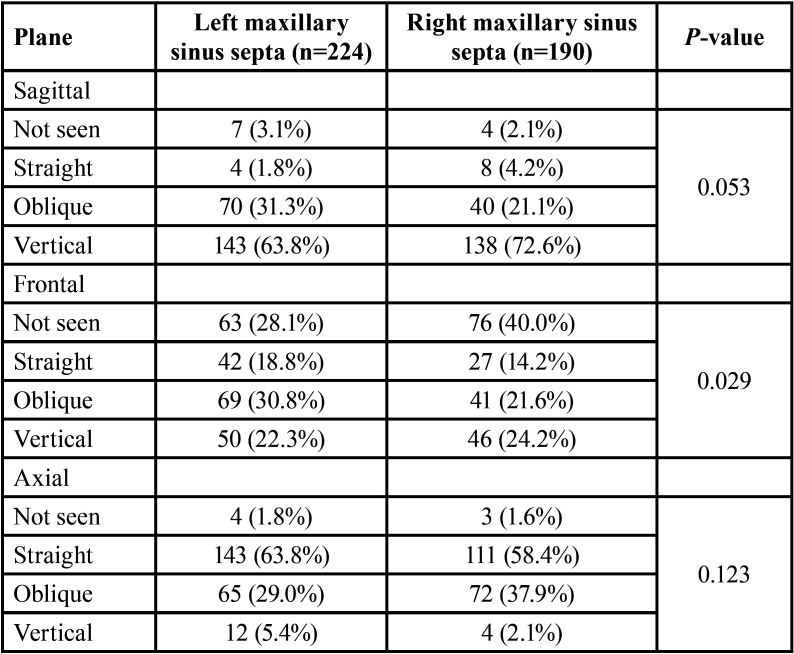
Distribution of septa direction in various planes of cone-beam computed tomography.

## Discussion

In this study, we tried to evaluate the prevalence of maxillary sinus septa, as well as their anatomical variations, based on the CBCT analysis. According to our findings, sinus septa were found in 37.9% of the subjects. Previous studies reported a variable prevalence of maxillary sinus septa ranging from 9.5% to 69% (12,13); for example, in a recently published study by Alhumaidan *et al*. (13), the authors reported that the prevalence of maxillary sinus septa was about 38% among the subject, which was similar to our study.

The results of the present study showed that there was a significant association between presence of septa and sex, that is, men had a higher number of septa versus women. This finding was consistent with some of the previous studies (12,14). It has been reported that men have a higher mean maximum bite force than women (15), which might explain the difference in the septa prevalence between men and women; however, some other studies did not report the same association (13,16). Concerning the association between age and septa prevalence, we found that septa were more frequently seen in younger cases than older age groups, which was in agreement with the study by Taleghani *et al*. (17), but inconsistent with some other surveys reporting a direct association between age and presence of septa (12,13).

Our results demonstrated that most of the septa were in the middle region of maxillary sinus, which was in agreement with most of the previous studies as mentioned in a systematic review by Malec *et al*. (18). We also observed that partial septa were much more prevalent than complete septa, which was consistent with previous papers reporting that most of the septa are partial (11,18). Regarding septa height, our findings indicated that the mean height of septa was higher in the left sinus than in the right sinus; these findings were in agreement with the results by Park *et al*. (19), but inconsistent with Al-Zahrani *et al*.’s study (12).

In the present survey, the visibility and orientation of septa in all three planes (i.e., frontal, sagittal, and axial) were also examined, resulting in useful information on each plane; for example, it was found that a considerable percentage of examined septa (33.6%) was not visible in the frontal plane, but it was observable in other two planes. Moreover, 16.7% of the septa found in our study straight direction in the frontal plane. This kind of septum may be very close to the sinus floor, which can appear like a thickness and mistakenly be considered as an additional border for the sinus floor, leading to mistakes in the measurement of the sinus floor thickness; thus, it seems necessary to check septa in the three different planes of CBCT.

The existence of septa should be considered for each sinus lifting surgery, because the presence of septa can cause perforation of the Schneiderian membrane and other postoperative complications. Also, there is a potential association between septa existence and the incidence of sinusitis in the maxilla (20,21). Schwarz *et al*. (22) investigated the risk factors of membrane perforation and postoperative complications in the sinus floor elevation surgery. They observed that membrane perforation was directly associated with the presence of sinus septa. Also, in the patients with membrane perforation, the odds of postoperative sinusitis was higher than those without.

CBCT provides cross-sectional images of the anatomical structures (such as the maxillary sinus) and can be used to exactly measure the dimensions and angles. This radiographic technique has the ability of data reconstruction, and due to the cursor-driven measurement algorithms, the clinicians can do real-time dimensional assessment. Considering these advantages, it is recommended to use CBCT to assess the sinus septa characteristics instead of panoramic view; however, more studies need to be conducted to compare the diagnostic performance of these two techniques for the anatomical variations of the sinus septa.

A limitation of this study was its retrospective designation, potentially leading to inaccessibility of some required information. Also, this study was performed in a single-center environment and our findings might not be generalized to other populations; therefore, it is proposed to overcome these limitations in the further investigations.

In conclusion, the results of the present study showed that the prevalence of the septa in the maxillary sinus was relatively high, with various directions, locations, heights, and shapes. It is recommended to use CBCT for a careful evaluation of toothless area prior to surgeries on the sinus (especially sinus lift) in three plans, in order to take careful measures and prevent postoperative complications in case septa exist.
